# The Protective Effect of Hispidin against Hydrogen Peroxide-Induced Oxidative Stress in ARPE-19 Cells via Nrf2 Signaling Pathway

**DOI:** 10.3390/biom9080380

**Published:** 2019-08-19

**Authors:** Sung-Ying Huang, Shu-Fang Chang, Siu-Fung Chau, Sheng-Chun Chiu

**Affiliations:** 1 Department of Ophthalmology, Hsinchu Mackay Memorial Hospital, Hsinchu 30071, Taiwan; 2 Department of Research, Taichung Tzu Chi Hospital, Buddhist Tzu Chi Medical Foundation, Taichung 42743, Taiwan; 3 Department of Ophthalmology, Taichung Tzu Chi Hospital, Buddhist Tzu Chi Medical Foundation, Taichung 42743, Taiwan; 4 Department of Laboratory Medicine, Taichung Tzu Chi Hospital, Buddhist Tzu Chi Medical Foundation, Taichung 42743, Taiwan; 5 General Education Center, Tzu Chi University of Science and Technology, Hualien 97005, Taiwan

**Keywords:** ARPE-19, hispidin, hydrogen peroxide, Nrf2, oxidative stress, age-related macular degeneration

## Abstract

Hispidin, a polyphenol compound isolated from *Phellinus linteus*, has been reported to possess antioxidant activities. In this study, we aimed to investigate the mechanisms underlying the protective effect of hispidin against hydrogen peroxide (H_2_O_2_)-induced oxidative stress on Adult Retinal Pigment Epithelial cell line-19 (ARPE-19) cells. Hispidin was not cytotoxic to ARPE-19 cells at concentrations of less than 50 μM. The levels of intracellular reactive oxygen species (ROS) were analyzed by dichlorofluorescin diacetate (DCFDA) staining. Hispidin significantly restored H_2_O_2_-induced cell death and reduced the levels of intracellular ROS. The expression levels of antioxidant enzymes, such as NAD(P)H:Quinine oxidoreductase-1 (NQO-1), heme oxygenase-1 (HO-1), glutamate-cysteine ligase catalytic subunit (GCLC), and glutamate-cysteine ligase modifier subunit (GCLM) were examined using real-time PCR and Western blotting. Our results showed that hispidin markedly enhanced the expression of nuclear factor erythroid 2-related factor 2 (Nrf2), HO-1, NQO-1, GCLM, and GCLC in a dose-dependent manner. Furthermore, knockdown experiments revealed that transfection with Nrf2 siRNA successfully suppresses the hispidin activated Nrf2 signaling in ARPE-19 cells. Moreover, activation of the c-Jun N-terminal kinase (JNK) pathway is involved in mediating the protective effects of hispidin on the ARPE-19 cells. Thus, the present study demonstrated that hispidin provides protection against H_2_O_2_-induced damage in ARPE-19 cells via activation of Nrf2 signaling and up-regulation of its downstream targets, including Phase II enzymes, which might be associated with the activation of the JNK pathway.

## 1. Introduction

Age-related macular degeneration (AMD) is the leading cause of blindness among the elderly in the developed world [1]. The most common type of AMD, also known as the dry-type or atrophic AMD, is initiated by changes in the pigmentation of the retinal pigment epithelial (RPE) cells and the sub-retinal deposits between the RPE and Bruch’s membrane. These progressions eventually result in RPE cell death, photoreceptors degeneration, and eventually loss of vision [2,3]. Unfortunately, there is no specific treatment available for dry/atrophic AMD.

Oxidative stress is characterized by the generation of reactive oxygen species (ROS), which plays a key role in the pathogenesis of AMD [4]. RPE cells have a high metabolic rate and exist in an environment which is abundant of endogenous ROS, such as O_2_^−^, H_2_O_2_ and OH·[5]. Long-term accumulation of oxidative damage leads to dysfunction in RPE cells and increases their susceptibility to oxidative stress. Several dietary supplements of antioxidants, including lutein, β-carotene, and vitamin C, appropriately alleviate the progression of AMD [6,7]. Thus, protecting RPE cells from oxidative stress is deemed to be a sustainable option for preventing the occurrence or decelerating the progression of AMD.

Nuclear factor erythroid 2-related factor 2 (Nrf2) is known for being a major regulator of the anti-oxidative responses which binds to the AREs (antioxidant response elements); thus, it plays a role in the up-regulation of the expression of antioxidant enzymes, including heme oxygenase-1 (HO-1), NAD(P)H:Quinine oxidoreductase-1 (NQO-1), glutamate-cysteine ligase catalytic subunit (GCLC), and glutamate-cysteine ligase modifier subunit (GCLM) [8,9]. Interestingly, a recent report shows that Nrf2-deficient mice present retinal pathology which is analogous to human AMD [10]. Recent studies also indicate that antioxidants work against oxidative stresses in RPE cells by activating Nrf2 signaling [11,12]. These findings suggest the key role of Nrf2 signaling in the protective effects of antioxidant on RPE cells.

*Phellinus linteus*, a species of fungus belonging to the genus *Phellinus* and the family *Hymenochaetaceae* has been extensively used as a medicinal mushroom in Africa and East Asia [13]. It has been reported to be rich in several polysaccharides and polyphenol compounds, including hispidin [14,15]. Hispidin is well-known for its antioxidant [15], anti-inflammatory [16,17], anti-proliferative, and anti-metastatic effects [18]. In addition, hispidin has been reported to function as an antioxidant agent by preventing the ROS associated damage in the pancreatic β-cells [19,20]. However, it still remains uncertain whether hispidin has the ability to protect the RPE cells from oxidative-stress-induced damage. In order to fill this gap in the existing knowledge, H_2_O_2_ was used to induce oxidative stress in the human RPE cell line, Adult Retinal Pigment Epithelial cell line-19 (ARPE-19)—which is isolated from human retinal pigmented epithelium—that has the structural and functional properties of RPE cells [21]. The purpose of this study was to determine the cytoprotective effect of hispidin on H_2_O_2_-induced oxidative stress in ARPE-19 cells and to investigate the association of Nrf2 signaling with the underlying mechanism.

## 2. Results

### 2.1. Effects of Hispidin on the Viability of ARPE-19 Cells

**Figure 1:**
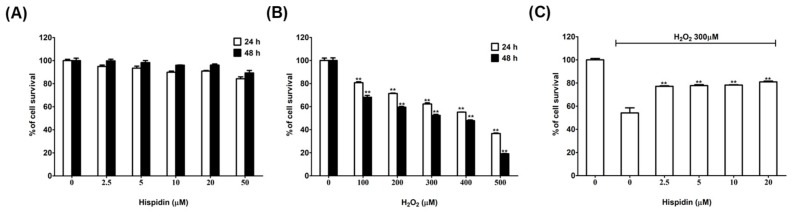
Effects of hispidin and H_2_O_2_ on the viability of Adult Retinal Pigment Epithelial cell line-19 (ARPE-19) cells. (**A**) ARPE-19 cells were treated with various concentrations (2.5–50 μM) of hispidin or (**B**) H_2_O_2_ (100–500 μM) for 24 (□) and 48 h (■), respectively. Cell survival was measured by MTT assay (**C**) ARPE-19 cells were pre-treated with hispidin (0–20 μM) for 24 h, followed by 300 μM H_2_O_2_ treatment for 24 h, cell survival was measured by MTT assay. ** *p* < 0.01 versus vehicle control.

To determine the optimal concentration without toxic effect of hispidin, ARPE-19 cells were treated with hispidin in various concentrations for 24 h or 48 h. The MTT assays revealed that there was no significant change in the viability of ARPE-19 cells after treated with hispidin at concentrations ranging from 2.5–50 μM (Figure 1A). Thus, this data indicates that hispidin is relatively non-toxic for use in ARPE-19 cells up to a concentration of 50 μM.

Using H_2_O_2_ to explore the protective effect against oxidative stress in RPE cells is a well-known model [22,23]. Thus, H_2_O_2_ was selected as the oxidative-stress inducer for our studies, and a working concentration of H_2_O_2_ that killed 50% of ARPE-19 cells after a 48 h incubation was determined by performing a dose-response experiment. The results showed that the viability of ARPE-19 cells decreased in a dose-dependent fashion in response to H_2_O_2_ treatment (Figure 1B). It was found that treatment with 300 μM H_2_O_2_ decreases cell viability by around 50% (52.4%); therefore, this concentration of H_2_O_2_ was selected for use in subsequent experiments.

To determine the protective effects of hispidin against H_2_O_2_-induced cell death on ARPE-19 cells, MTT assays were performed. The results showed that treatment with 300 μM H_2_O_2_ led to a significant reduction in cell viability (by 54.2%) as compared with the control cells; whereas, pre-treatment with hispidin (2.5–20 μM) for 24 h resulted in the prevention of H_2_O_2_-induced cell death (Figure 1C). Furthermore, pre-treatment of ARPE-19 cells with 20 μM hispidin restored the cell viability up to 80.9% with respect to the untreated cells. These results suggest that hispidin can help protect ARPE-19 cells from H_2_O_2_-induced cell death.

### 2.2. Hispidin Protects ARPE-19 Cells Against H_2_O_2_-Induced Oxidative Stress

**Figure 2:**
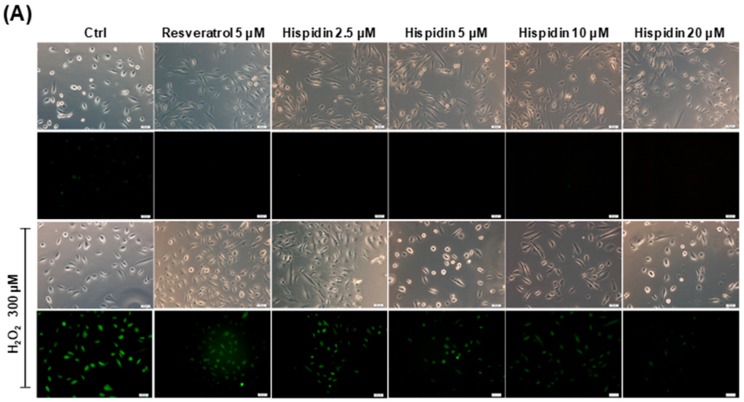

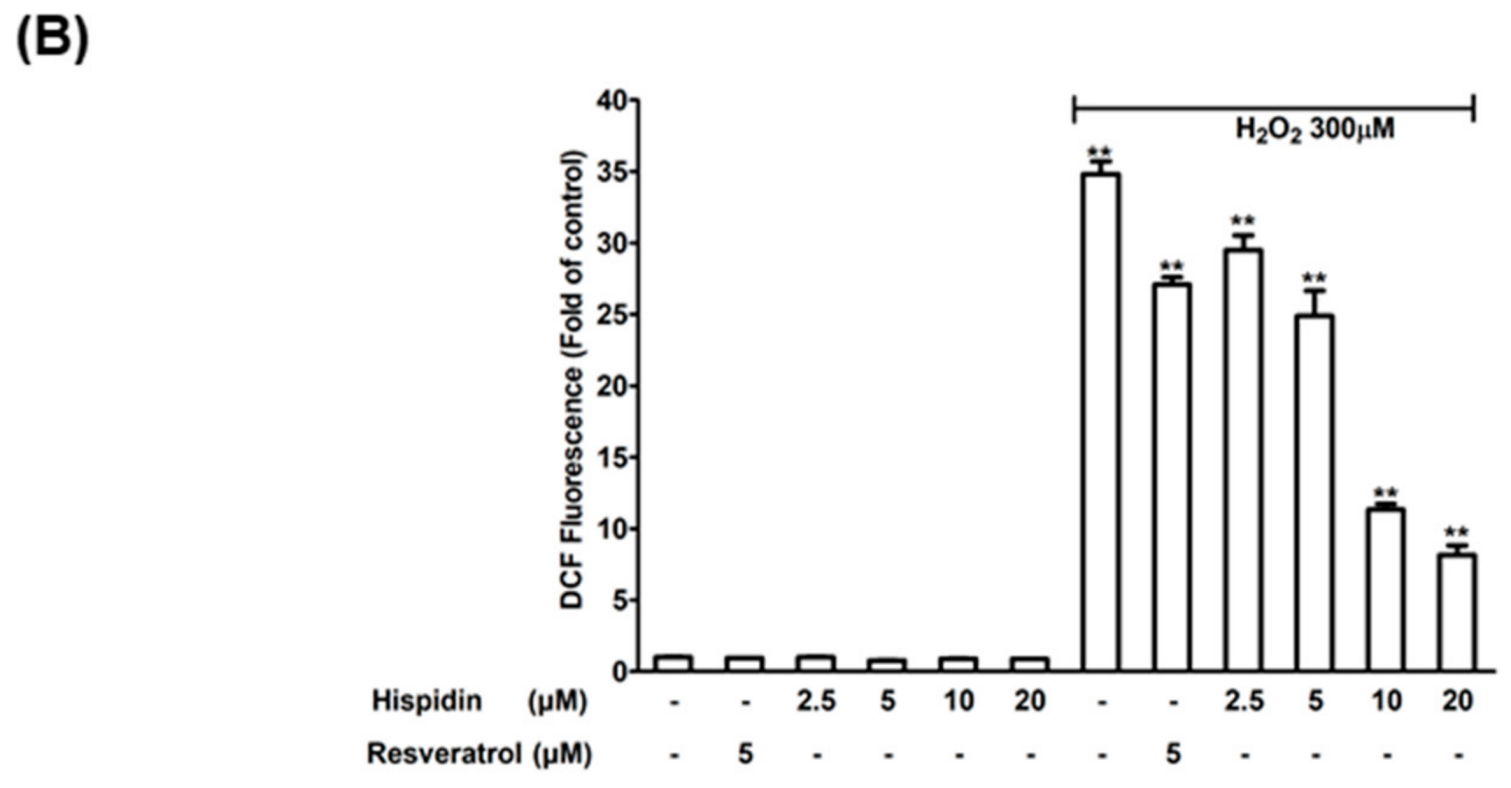
Protective effect of hispidin against H_2_O_2_-induced oxidative stress on ARPE-19 cells. (**A**) ARPE-19 cells were pre-treated with various concentrations (2.5–20 μM) of hispidin for 24 h and then treated with 300 μM H_2_O_2_ for 6 h. The H_2_O_2_-induced reactive oxygen species (ROS) generation was measured by dichlorofluorescin diacetate (DCFDA) assay. The green fluorescence represents the cells stained for ROS (magnification 200x); (**B**) Fluorescence was then measured with a multi-well fluorescence reader at 535 nm (emission). ** *p* < 0.01 versus vehicle control.

Hispidin has been reported to possess quenching effects against free radicals. To evaluate the ROS scavenging ability of hispidin on ARPE-19 cells, dichlorofluorescin diacetate (DCFDA) assay was performed. The fluorescence microscopy results revealed that the levels of ROS in 300 μM H_2_O_2_-treated cells were enhanced as compared to the vehicle group (Figure 2A). However, pre-treatment with hispidin (2.5–20 μM) for 24 h prominently decreased the fluorescence intensity as compared to the H_2_O_2_-only group. The fluorescence signal at 535 nm was measured by a fluorescence plate reader (Figure 2B). Cells treated with 300 μM H_2_O_2_ showed a 34.8-fold induction of intracellular ROS as compared to the non-treated group. However, pre-treatment with hispidin at concentrations of 2.5 μM, 5 μM, 10 μM, and 20 μM significantly reduced the intracellular ROS to 29.5-, 24.9-, 11.3-, and 8.2-fold, respectively. Cells treated with 5 μM resveratrol as a positive control showed a 27.1-fold induction in the concentration of intracellular ROS. These results indicate that hispidin reduces H_2_O_2_-induced intracellular ROS in a dose-dependent manner.

### 2.3. Hispidin Activates Nrf2 and Its Target Genes Involved in Anti-Oxidative Response in ARPE-19 Cells

**Figure 3:**
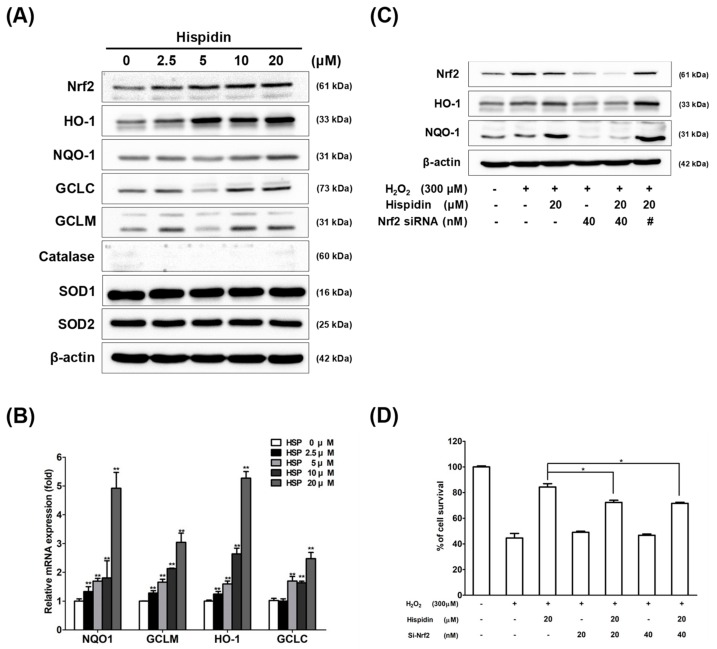
Activation of nuclear factor erythroid 2-related factor 2 (Nrf2) signaling is corelated with the protective effect of hispidin against H_2_O_2_-induced cell death on ARPE-19 cells. (**A**) Cells were treated with various concentrations (2.5–20 μM) of hispidin for 24 h, and the expression levels of Nrf2, heme oxygenase-1 (HO-1), NAD(P)H:Quinine oxidoreductase-1 (NQO-1), glutamate-cysteine ligase catalytic subunit (GCLC), glutamate-cysteine ligase modifier subunit (GCLM), catalase, superoxide dismutase 1 (SOD1), and superoxide dismutase 2 (SOD2) were analyzed by Western blot or real-time PCR (**B**). (**C**) Cells were transfected with 40 μM Nrf2 siRNA or scramble siRNA (#) for 48 h, and then pre-treated with hispidin for 24 h, followed by 24 h H_2_O_2_ treatment. Protein levels of Nrf2, HO-1, and NQO-1 were analyzed by Western blot, and cell viability was assessed by MTT assay (**D**). * *p* < 0.05 versus hispidin+H_2_O_2_ group.

To further elucidate whether the activation of antioxidant enzymes is involved in the ROS scavenging activities of hispidin, ARPE-19 cells were treated with various concentrations of hispidin for 48 h, and the protein-expression levels of antioxidants, including Nrf2, HO-1, NQO-1, GCLC, GCLM, catalase, and superoxide dismutase (SOD, SOD1:Cu/ZnSOD, and SOD2:MnSOD), were examined by Western blot (Figure 3A). The expression of Nrf2, HO-1, NQO-1, GCLC, and GCLM were up-regulated after treatment with hispidin. However, the expression of catalase, SOD1, and SOD2 did not increase on treatment with hispidin. Consistent with the results of the Western blot, all the indicated concentrations of hispidin (2.5–20 μM) treatment increased the expression of NQO-1, GCLM, HO-1, and GCLC, transcripts as assessed by real-time PCR analysis (Figure 3B). These results demonstrated that the expression of Nrf2 and its downstream target genes, such as NQO-1, HO-1, GCLC, and GCLM, were up-regulated after hispidin treatment in ARPE-19 cells.

In order to determine the role of Nrf2 in mediating the protective effects of hispidin on RPE cells, the siRNA approach was employed. Cells were transfected with 40 nM Nrf2 siRNA or control scramble siRNA for 48 h, and then pre-treated with or without 20 μM hispidin for 24 h, followed by treatment with 300 μM H_2_O_2_ for 24 h (Figure 3C). Immunoblotting analysis revealed that transfection with Nrf2 siRNA suppressed the hispidin-induced Nrf2 expression in ARPE-19 cells, as compared with the vehicle group (scrambled siRNA transfected). The down-regulated expression of the Nrf2 downstream target genes HO-1 and NQO-1 were also observed. In addition, upon transfection with Nrf2 siRNA, the cell viability after H_2_O_2_ treatment was attenuated from 84.3% to 72.3% (20 nM) or 71.5% (40 nM), as measured by MTT assays (Figure 3D). Taken together, these results indicate that hispidin-induced protective effects against H_2_O_2_ are due to the up-regulation of Nrf2 and its downstream target genes in ARPE-19 cells.

### 2.4. Hispidin Induces C-Jun N-Terminal Kinase (JNK) Activation Involved in Anti-Oxidative Response in ARPE-19 Cells

**Figure 4:**
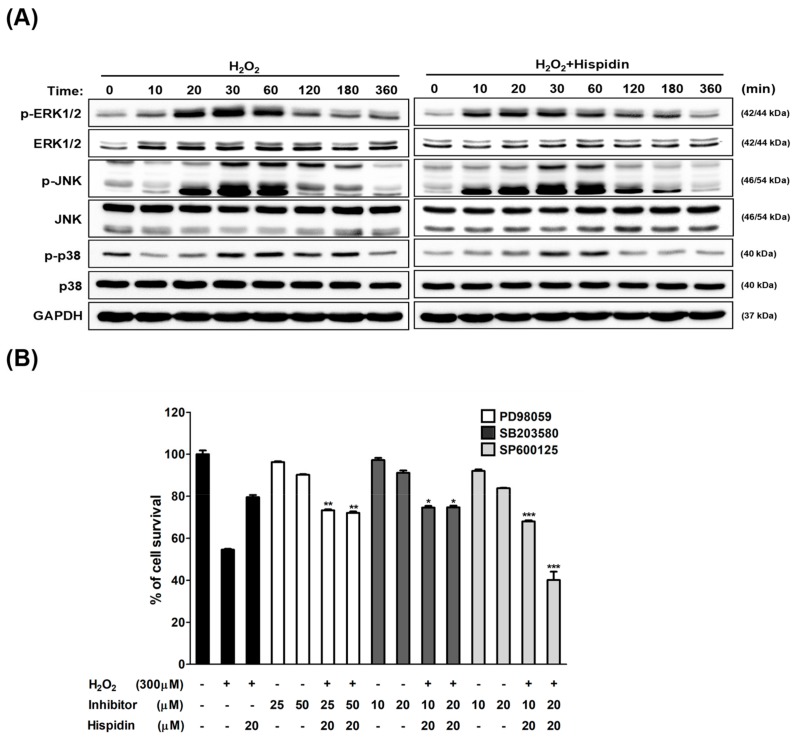
Hispidin induces c-Jun N-terminal kinase (JNK) activation in ARPE-19 cells exposed to H_2_O_2_-induced oxidative stress. (**A**) ARPE-19 cells were treated with or without hispidin (20 μM) for 24 h and then treated with 300 μM H_2_O_2_ for 10–360 min. Proteins were collected and subjected to analyze the expression levels of p-ERK, p-JNK, and p-p38, which were then evaluated by Western blot. Glyceraldehyde 3-phosphate dehydrogenase (GAPDH) was used as an internal control. (**B**) ARPE-19 cells were treated with hispidin for 24 h in the presence or absence of the mitogen-activated protein kinase (MAPK) pathway inhibitors, followed by a 300 μM H_2_O_2_ treatment for 24 h. Cell viability was analyzed by MTT assay. * *p* < 0.05, ** *p* < 0.01, *** *p* < 0.001 versus hispidin+H_2_O_2_ group.

To determine the roles of mitogen-activated protein kinase (MAPK) signaling involved in the protective effect of hispidin on ARPE-19 cells, cells were pre-treated with 20 μM hispidin for 24 h and then treated with 300 μM H_2_O_2_ for 10–360 min. After analyzed by Western blot, protein levels of phospho-JNK in cells treated with hispidin were augmented during 10–180 min post H_2_O_2_ treatment as compared to the H_2_O_2_ alone group (Figure 4A). To further elucidate how the JNK pathway is associated with the protective effects of hispidin against oxidative stress, cells were treated with 20 μM hispidin for 24 h in the presence or absence of the following MAPK signaling inhibitors: MEK1/2 inhibitor PD98059 (25 μM or 50 μM), the p38 inhibitor SB203580 (10 μM or 20 μM), or the JNK inhibitor SP600125 (10 μM or 20 μM). Then cells were subjected to a 300 μM H_2_O_2_ treatment for 24 h, followed by MTT assay analysis (Figure 4B). The results showed that only SP600125 treatment can significantly reduce the cell viability to 72.7% (10 μM) and 40.1% (20 μM) as compared to the vehicle control, which suggested that treatment with JNK but not MEK1/2 or p38 inhibitor abolished the protective effects of hispidin in H_2_O_2_-treated ARPE-19 cells. These results indicate that the activation of the JNK pathway plays a crucial role in mediating the protective effect of hispidin on H_2_O_2_-induced cell death in ARPE-19 cells.

### 2.5. JNK Pathway is Essential for Hispidin-Induced Activation of Nrf2 Signaling in ARPE-19 Cells

**Figure 5:**
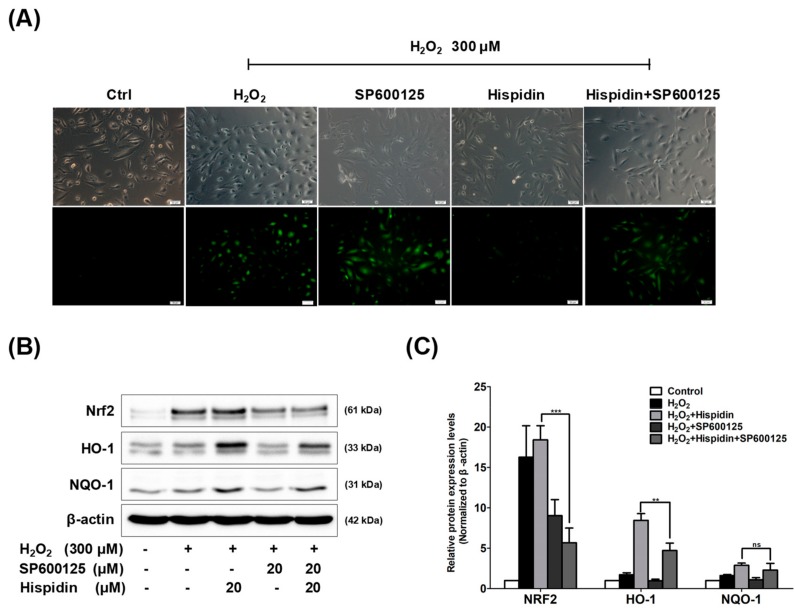
The role of JNK activation in hispidin-induced Nrf2 signaling on ARPE-19 cells. (**A**) Cells were pre-treated with 20 μM hispidin, 20 μM SP600125, or both for 24 h and then treated with 300 μM H_2_O_2_ for 6 h. The ROS generation was measured by dichlorofluorescin diacetate (DCFDA) assay. The green fluorescence represents the cells stained for ROS (magnification 200×). (**B**) Cells were pre-treated with 20 μM hispidin or 20 μM SP600125 for 24 h followed by 300 μM H_2_O_2_ treatment for 24 h. The expression levels of Nrf2, HO-1, and NQO-1 were evaluated by Western blot. (**C**) Quantitative analysis was performed by measuring the intensity relative to the control. ** *p* < 0.01, *** *p* < 0.001, ns: not significant.

To elucidate the possible role of JNK activation in the hispidin-induced antioxidant effect, ARPE-19 cells were treated with hispidin (20 μM) for 24 h in the presence and absence of the SP600125 (20 μM), then treated with 300 μM H_2_O_2_ for 6 h, followed by dichlorofluorescin diacetate (DCFDA) staining (Figure 5A). The data showed that the fluorescence intensity was enhanced in the H_2_O_2_- or SP600125-alone groups, but not in the hispidin pre-treatment group. However, treatment with SP600125 significantly enhanced the fluorescence intensity in the hispidin pre-treatment group. The involvement of JNK activation in the regulation of Nrf2 signaling was also investigated. ARPE-19 cells were treated with hispidin (20 μM) for 24 h in the presence and absence of the SP600125 (20 μM), followed by 300 μM H_2_O_2_ treatment for 24 h, and then subjected to Western blot analysis (Figure 5B). The protein expression levels of Figure 5B were quantified and normalized to the β-actin (Figure 5C). The results revealed that SP600125 significantly blocks the hispidin-induced expression of genes such as Nrf2 and HO-1, as compared to the indicated group. Thus, the overall data suggests that the activation of the JNK pathway is critical for the hispidin-induced antioxidant effect and the activation of Nrf2 signaling.

## 3. Discussion

Hispidin has been demonstrated to protect pancreatic β-cells from H_2_O_2_-induced damage through ROS scavenging activity [19]. Tu et al. have reported that hispidin inhibits ROS and nitric oxide (NO) production in adipocytes [24]. Park et al. have demonstrated the cytoprotective effect of hispidin on myotubes from oxidative-stress-induced injury by inhibiting oxidative stress and suppressing apoptosis [25]. However, the effects of hispidin on RPE cells to protect against H_2_O_2_-induced oxidative stress is not elucidated yet. Thus, the aim of the present study is to analyze the antioxidant effects of hispidin on H_2_O_2_-induced oxidative stress in RPE cells and to determine its underlying molecular mechanism of action. In this study, an MTT assay showed that hispidin does not have a toxic effect on ARPE-19 cells at concentrations less than 50 μM. Pre-treatment of hispidin significantly increases the viability of ARPE-19 cells from H_2_O_2_-induced oxidative injury. The increased cell viability in response to 2.5 μM was similar to that of 5 μM, 10 μM, and 20 μM. However, as the protective effect of hispidin against oxidative stress was significantly better at 20 μM, this concentration of hispidin was determined to be optimal for subsequent experiments in this study. These results indicate that hispidin exerts a cytoprotective effect on ARPE-19 cells exposed to H_2_O_2_-induced oxidative stress by enhancing cell viability. The chemical structure of hispidin is very similar to resveratrol, which is well-known as a strong antioxidant [26]. In the present study, hispidin, as well as resveratrol pre-treatment, reduced ROS production, as observed by DCFDA staining. Several antioxidant enzymes, including SOD, protect against ROS-induced damage in RPE cells [27]. However, hispidin up-regulated the expression of HO-1 and NQO-1, but not that of SODs (SOD1, SOD2, and catalase) in H_2_O_2_-treated ARPE-19 cells. These results suggest that the protective effects of hispidin are due to its role as a ROS scavenger, whereby it enhances the expression of antioxidant enzyme, thereby attenuating the oxidative damage.

Upon further investigating the possible pathways involved in mediating the cytoprotective ability of hispidin against oxidative stress in ARPE-19 cells, it was revealed that the downstream target genes of Nrf2-signaling HO-1 and NQO-1, play crucial roles in protecting the cells from oxidative stress [28,29]. Recent reports have indicated the beneficial effects of Nrf2 signaling on RPE cells [30,31]. Various studies have demonstrated that the activation of Nrf2/HO-1 signaling is essential for the reduction of the oxidative damage to RPE cells [32,33,34]. In this study, it was proposed that the anti-oxidative effects of hispidin might incorporate with Nrf2 signaling. Our data demonstrates that hispidin protects the ARPE-19 cells from H_2_O_2_-induced oxidative damage by activating the Nrf2-signaling pathway and inducing the expression of Nrf2, HO-1, NQO-1, GCLC, and GCLM. In addition, Nrf2 silencing attenuates the protective effects of hispidin and suppresses the expression of its downstream targets, HO-1 and NQO-1.

In the current study, activation of the JNK pathway appeared to be involved in the protective effect of hispidin against H_2_O_2_-induced oxidative stress. Our findings have revealed that hispidin treatment induces significant JNK phosphorylation, whereas treatment with a JNK inhibitor (SP600125) reduces the JNK phosphorylation and attenuates the cytoprotective effect of hispidin against H_2_O_2_ in ARPE-19 cells. Moreover, the ROS scavenging activity of hispidin was found to be abolished by SP600125 pre-treatment. Besides, hispidin-induced Nrf2/HO-1 expression was directly regulated by the JNK pathway. In addition, treatment with SP600125 reduced the hispidin-induced protein expression such as Nrf2, HO-1, and NQO-1. Therefore, to our knowledge, our study has for the first time demonstrated that naturally occurring hispidin isolated from *P. linteus* suppresses cellular damage and oxidative stress induced by H_2_O_2_ via JNK-Nrf2 dependent HO-1 expression in ARPE-19 cells.

The blood-retina barrier (BRB) is a physiological barrier that regulating material transport between the retina and circulating blood [35]. The development of the drug delivery system across the BRB is widely expected to improve the treatment of retinal diseases such as AMD and diabetic retinopathy. Since positively charged molecule could penetrate across the BRB [36,37], hispidin could be formulated into either cationic macromolecule or nanoparticles for further in vivo application. The cationic bio-degradable polymers are ideal candidates for hispidin formulation. Naturally derived biocompatible polymers, including chitosan, gelatin, dextran, and cellulose, were not only applied in gene delivery but also for various therapeutic purpose [38]. Besides, various phospholipid-based nano-formulated drugs have been approved in clinical use [39]. The cationic lipid was also applied to encapsulate the p53 gene for delivery and restoring the wild-type p53 protein in cancer treatment. According to the relative research, a cationic formulated particle system exhibits great potential for hispidin application, and the in vivo experiments are worthy of further investigation.

## 4. Conclusion

**Figure 6:**
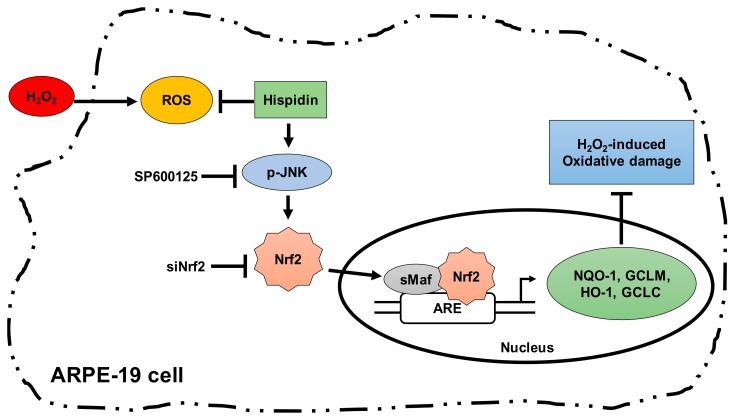
Schematic representation a proposed pathway for hispidin-induced Nrf2/ARE-mediated cytoprotective proteins. Up-regulation of Nrf2 target genes such as HO-1 explains the protective effects against H_2_O_2_-induced oxidative stress in ARPE-19 cells. ARE: Antioxidant response elements; sMaf: Small Maf (musculoaponeurotic fibrosarcoma).

In conclusion, the present study presents the novel functions of hispidin that are responsible for protection of ARPE-19 cells against H_2_O_2_-induced oxidative damage by suppressing ROS levels. To our knowledge, our data has demonstrated for the first time that hispidin suppresses H_2_O_2_-induced oxidative stress and cell death through induction of Nrf2/HO-1 expression via a JNK-Nrf2-dependent pathway in ARPE-19 cells (Figure 6). These results suggest that hispidin has the potential to serve as a therapeutic candidate for AMD treatment or prevention.

## 5. Materials and Methods

### 5.1. ARPE-19 Cells

The ARPE-19 cell line was purchased from BCRC (Bioresource Collection and Research Center, Hsinchu, Taiwan), authenticated by short-tandem repeat analysis, and cultured in its standard medium, as recommended by the BCRC. The culture medium, fetal bovine serum, and supplements were all purchased from Invitrogen, Carlsbad, CA, USA. For cell viability and DCFDA assays, ARPE-19 cells were seeded at a density of 3 × 10^4^ in 24-well plates. For real-time PCR and Western blot analyses, cells were seeded at a density of 3 × 10^5^ in a 6 cm culture petri dish.

### 5.2. Chemicals and Reagents

The hispidin was first isolated from *Polyporus hispidulus,* and the production and structure elucidation came from the mycelial broth of *Phellinus linteus* [15]. It can also be synthesized [40]. In this study, hispidin (C_13_H_10_O_5_, 98%, soluble in DMSO at 20 mg/mL), MTT, and DCFDA were purchased from Sigma Chemical Co. (St. Louis, MO, USA). The Nrf2 (ab52352) antibody was purchased from Abcam (Cambridge, MA, USA), and all other antibodies were purchased from GeneTex Inc. (San Antonio, TX, USA).

### 5.3. Cell Viability Assay

The viability of the cells was evaluated using MTT assay, as described [41,42].

### 5.4. DCFDA Assay

ROS were detected using 2′,7′-dichlorofluorescein diacetate (DCFDA) assay. Cells were loaded with 5 μM DCFDA, followed by incubation for 6 h. Fluorescence signal at 535 nm (excitation at 482 nm) was measured using an Infinite 200 Pro TecanTM (Tecan, Mannedorf, Switzerland). The background fluorescence signal was measured immediately after addition of reagent.

### 5.5. Western Blot Analysis

Western blot analysis was performed as described [41,42].

### 5.6. Real-Time RT-PCR Analysis

Real-time RT-PCR analysis was performed as described [41,42]. Real-time RT-PCR primer sequences used in this study were GCLC F-5′-AAGCCATTCACTCCAGATTTTACC-3′, GCLM F-5′-ACTGACTTAGGAGCATAACTTACC-3′, GAPDH F-5′-CCATGGAGAAGGCTGGGG -3′, R-5′-CAAAGTTGTCATGGATGACC -3′, R-5′-AAGAATATCTGCCTCAATGACACC-3′, HO-1 F-5′- ATGACACCAAGGACCAGAGC-3′, R-5′- GTAAGGACCCATCGGAGAAGC-3′, R-5′-ACAACAAACTTCAACGCAAAGC-3′, NQO1 F-5′-TATCCTGCCGAGTCTGTTCTG-3′, and R-5′-AACTGGAATATCACAAGGTCTGC-3′.

### 5.7. Small Interfering RNA (siRNA) Transfection

For siRNA transfection, ARPE-19 cells were transfected with 20 or 40 nM siRNA, using RNAifect Transfection Reagent (QIAGEN) and analyzed 48 h post-transfection. Nrf2 siRNA were purchased from Dhamarcon RNAi Technologies (Lafayette, CO, USA). ON-TARGETplus SMARTpool Nrf2 siRNA sequences: (1) UAAAGUGGCUGCUCAGAAU, (2) GAGUUACAGUGUCUUAAUA, (3) UGGAGUAAGUCGAGAAGUA, and (4) CACCUUAUAUCUCGAAGUU.

### 5.8. Statistical Analysis

All data were analyzed using the Student’s *t* test for normally distributed values and by the nonparametric Mann–Whitney U test for values with a non-normal distribution as described [41,42].
